# Supplementary material for: Simulation platform for pattern recognition based on reservoir computing with memristor networks
Source: Sci Rep. 2022 Jun 14;12:9868. doi: 10.1038/s41598-022-13687-z (PMC9197854; doi:10.1038/s41598-022-13687-z)
Supplement: Supplementary file 1 — Supplementary Information 1. [file 41598_2022_13687_MOESM1_ESM.pdf]

Supplementary Information for

Simulation platform for pattern recognition based on reservoir computing with memristor networks

Gouhei Tanaka[1,2,3,*] and Ryosho Nakane[2]

[1] International Research Center for Neurointelligence, The University of Tokyo, Tokyo 113-0033, Japan

[2] Department of Electrical Engineering and Information Systems, Graduate School of Engineering, The University of Tokyo, Tokyo 113-8656, Japan

[3] Department of Mathematical Informatics, Graduate School of Information Technology and Science, The University of Tokyo, Tokyo 113-8656, Japan

*Correspondence to: gtanaka@g.ecc.u-tokyo.ac.jp

This file includes:

Supplementary Figures 1-7

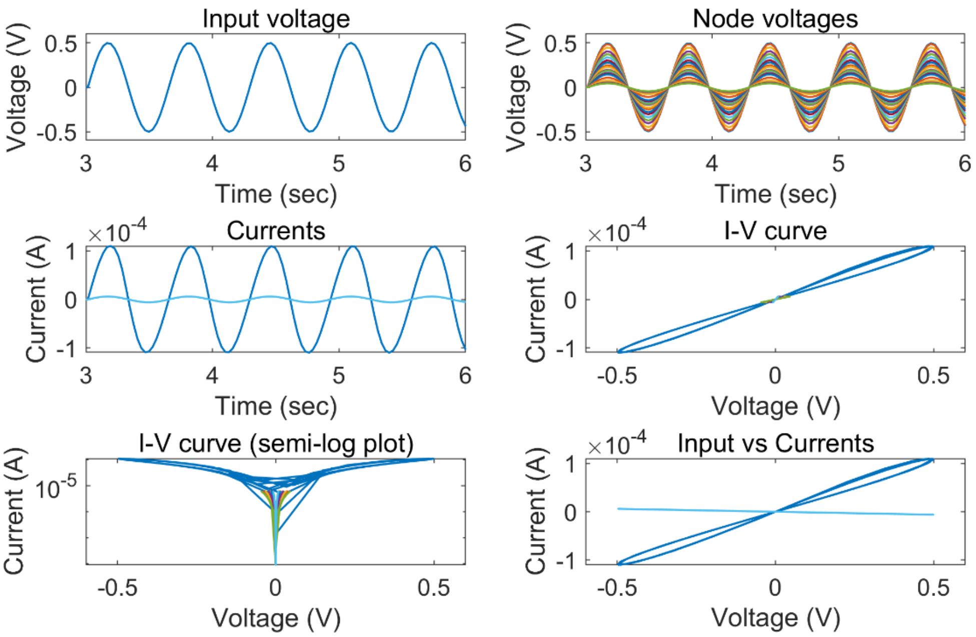

Supplementary Figure 1: Dynamical behavior of memristor-network-based reservoir driven by a sinusoidal input. The same as Figure 1, but for a network of the Ring-UP type.

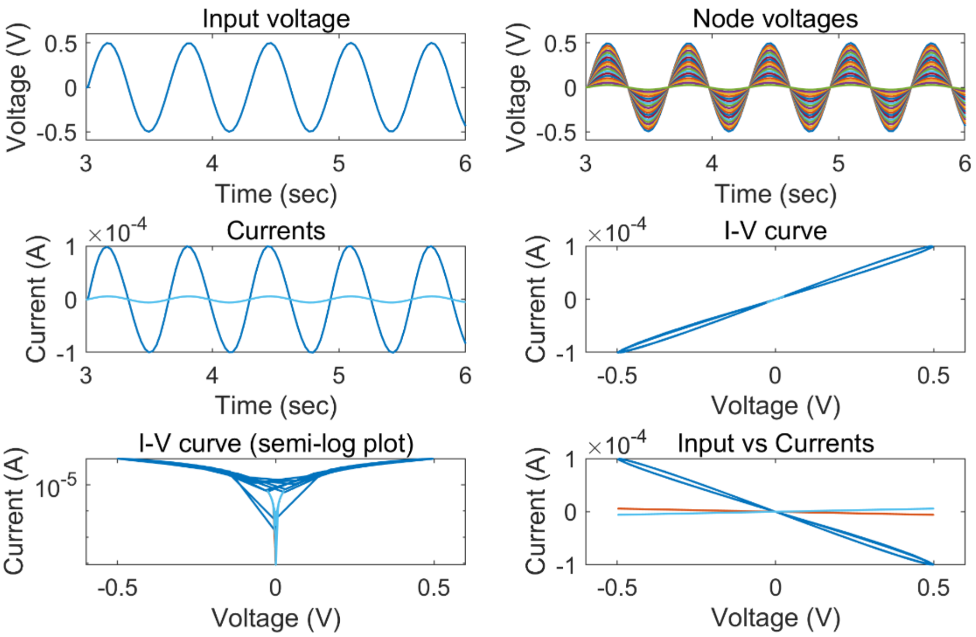

Supplementary Figure 2: Dynamical behavior of memristor-network-based reservoir driven by a sinusoidal input. The same as Figure 1, but for a network of the Ring-RP type.

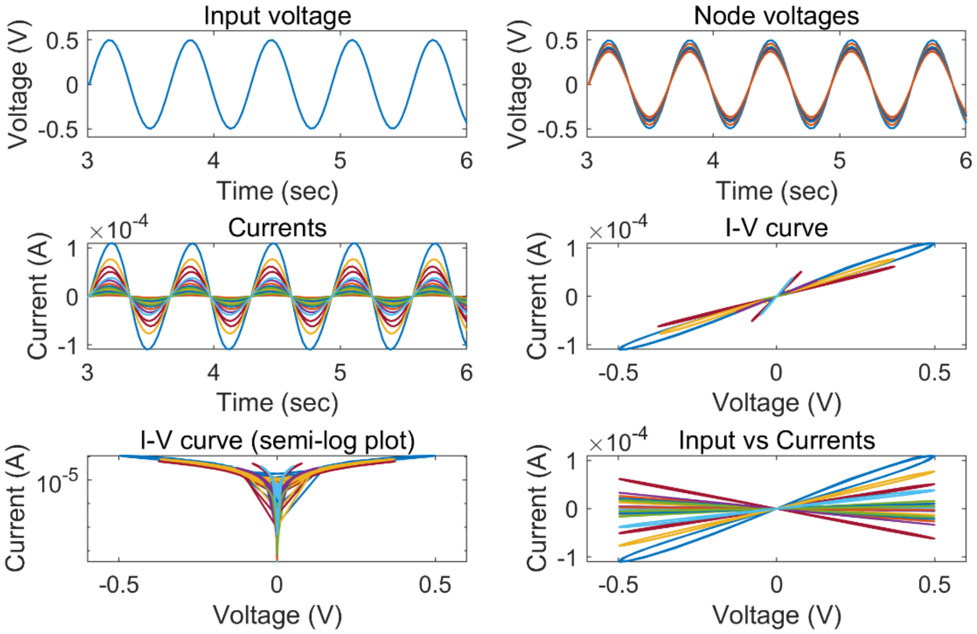

Supplementary Figure 3: Dynamical behavior of memristor-network-based reservoir driven by a sinusoidal input. The same as Figure 1, but for a network of the Rand-RP type.

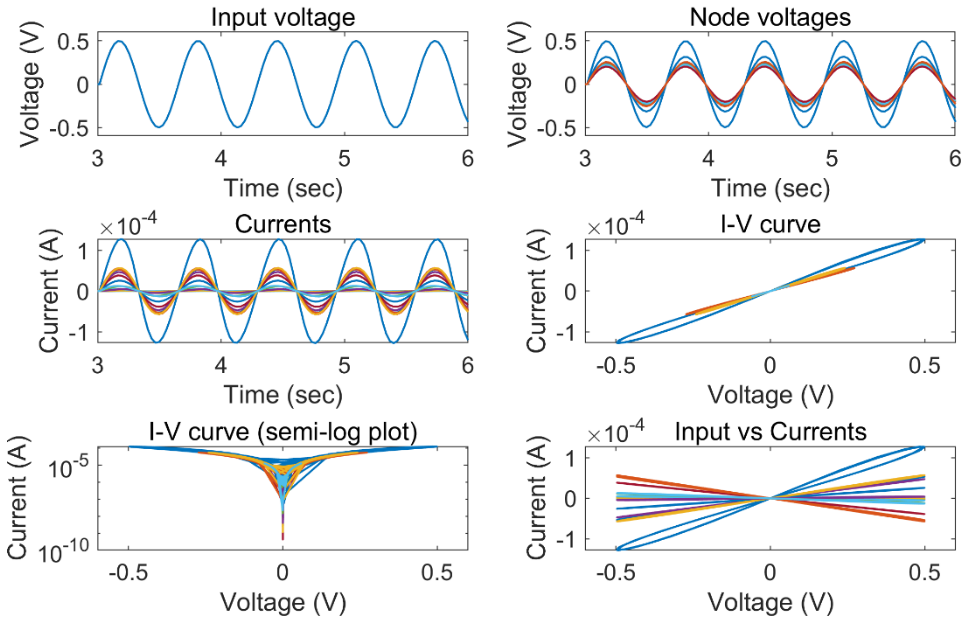

Supplementary Figure 4: Dynamical behavior of memristor-network-based reservoir driven by a sinusoidal input. The same as Figure 1, but for the variability parameter set at $\sigma = 0$.

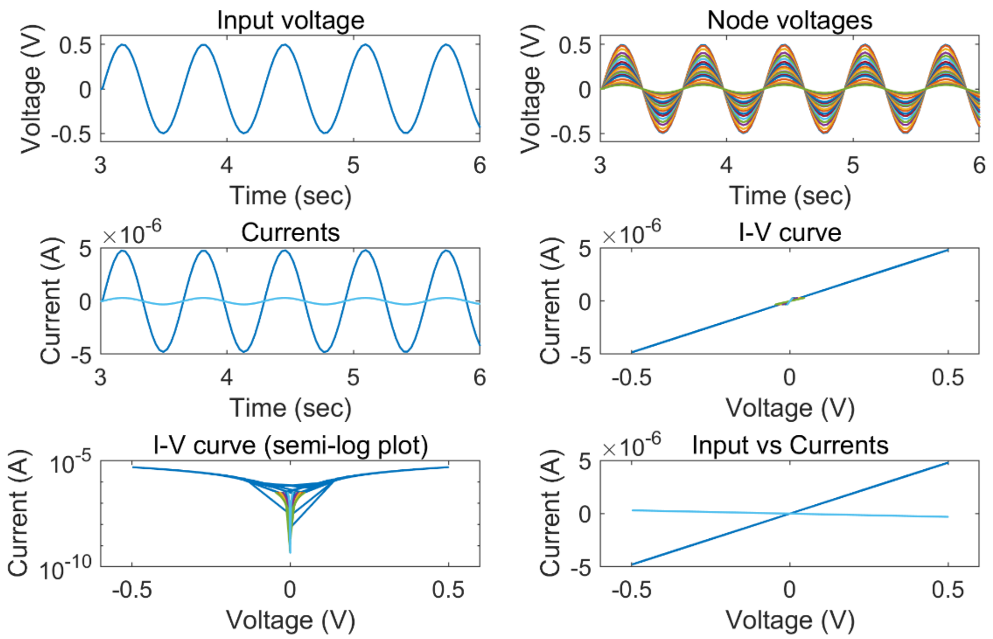

Supplementary Figure 5: Dynamical behavior of memristor-network-based reservoir driven by a sinusoidal input. The same as Figure 1, but for the nonlinearity parameter set at $\bar{r} = 10^3$.

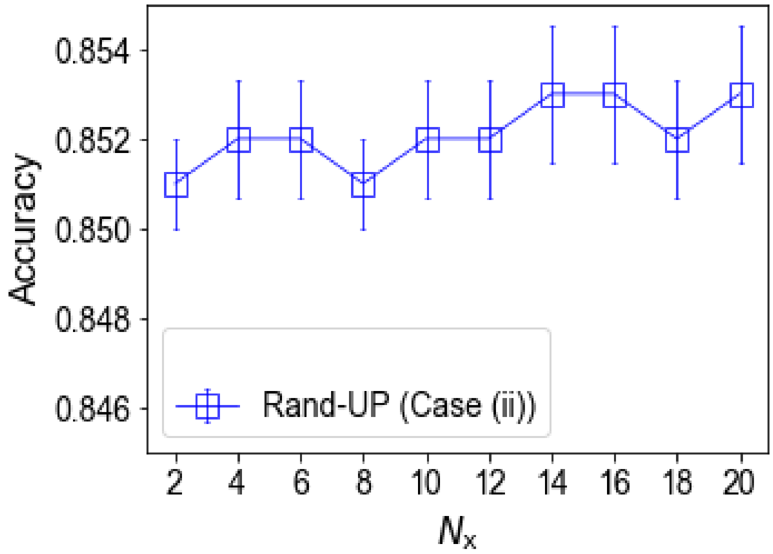

Supplementary Figure 6: Effect of the number of signals used for the readout in the ECG classification task. The enlargement of Figure 5c, showing that the performance is slightly improved by increasing N_x (only for Rand-UP (Case (ii)).

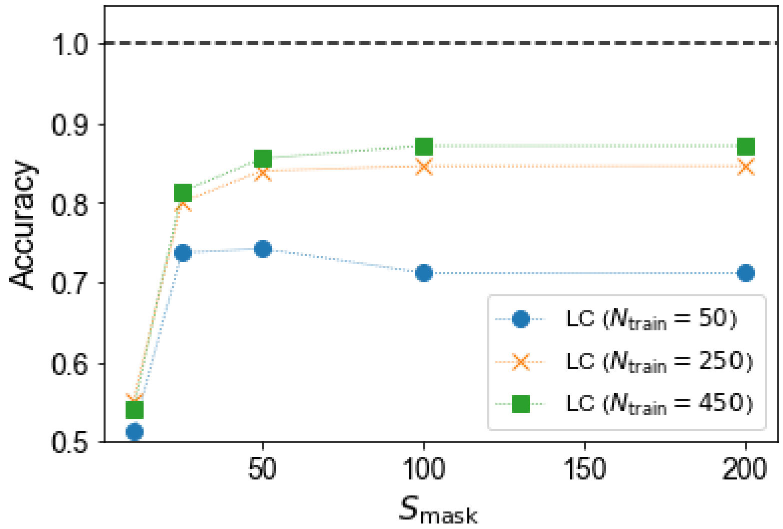

Supplementary Figure 7: The effect of mask size on the performance of the linear classifier. The accuracy peaks out at around $S_{\mathrm{mask}} = 100$.
